# Computational Method for Quantitative Comparison of Activity Landscapes on the Basis of Image Data

**DOI:** 10.3390/molecules25173952

**Published:** 2020-08-29

**Authors:** Javed Iqbal, Martin Vogt, Jürgen Bajorath

**Affiliations:** Department of Life Science Informatics, B-IT, LIMES Program Unit Chemical Biology and Medicinal Chemistry, Rheinische Friedrich-Wilhelms-Universität, Endenicher Allee 19c, D-53115 Bonn, Germany; jiqbal@bit.uni-bonn.de (J.I.); martin.vogt@bit.uni-bonn.de (M.V.)

**Keywords:** active compounds, three-dimensional activity landscapes, topological features, structure–activity relationships, image analysis, grid representations, landscape comparison, similarity measures

## Abstract

Activity landscape (AL) models are used for visualizing and interpreting structure–activity relationships (SARs) in compound datasets. Therefore, ALs are designed to present chemical similarity and compound potency information in context. Different two- or three-dimensional (2D or 3D) AL representations have been introduced. For SAR analysis, 3D AL models are particularly intuitive. In these models, an interpolated potency surface is added as a third dimension to a 2D projection of chemical space. Accordingly, AL topology can be associated with characteristic SAR features. Going beyond visualization and a qualitative assessment of SARs, it would be very helpful to compare 3D ALs of different datasets in more quantitative terms. However, quantitative AL analysis is still in its infancy. Recently, it has been shown that 3D AL models with pre-defined topologies can be correctly classified using machine learning. Classification was facilitated on the basis of AL image feature representations learned with convolutional neural networks. Therefore, we have further investigated image analysis for quantitative comparison of 3D ALs and devised an approach to determine (dis)similarity relationships for ALs representing different compound datasets. Herein, we report this approach and demonstrate proof-of-principle. The methodology makes it possible to computationally compare 3D ALs and quantify topological differences reflecting varying SAR information content. For SAR exploration in drug design, this adds a quantitative measure of AL (dis)similarity to graphical analysis.

## 1. Introduction

Graphical representations are desirable to support the analysis of structure–activity relationships (SARs), especially when large sets of active compounds are investigated [1,2]. SARs are determined by chemical similarity and potency relationships between compounds active against a given target [3]. If sequential structural modifications of compounds lead to small or moderate changes in potency, SARs are continuous in nature. By contrast, if small structural changes cause potency alterations of large magnitude, SARs are discontinuous. Activity cliffs (ACs), i.e., pairs of structural analogs with large potency differences, are centers of SAR discontinuity in datasets [3,4]. Activity landscape (AL) representations were introduced to combine the analysis of similarity and potency information in various ways [5,6,7]. AL representations differ substantially in their design and complexity [5]. They range from plots and graphs such as the structure–activity similarity map [8,9], SAR map [10], or ligand-target differentiation map [11] and annotated molecular networks such as network-like similarity graphs [12] to multi-dimensional representations [13]. Formally, in an *n*-dimensional AL model, *n*-1 dimensions represent a chemical descriptor/feature space and the *n*th dimension represents activity space [13]. In other words, a multi-dimensional AL can be rationalized as a chemical feature space containing a biological activity hypersurface [5]. For SAR visualization and interpretation, three-dimensional (3D) AL views are particularly attractive because they are akin to geographical maps, with topologies accounting for the presence of characteristic local SARs [13,14,15]. Accordingly, smooth regions in 3D ALs mirror SAR continuity, whereas rugged regions are indicative of SAR discontinuity and contain ACs. For different compound datasets, 3D AL models can be constructed by combining a two-dimensional (2D) projection of (*n*-1)-dimensional chemical space with compound potency values subsequently added as a third dimension [14,15]. From distributed potency values, a coherent potency surface is interpolated and color-coded by potency, resulting in a 3D view reminiscent of a geographical map [14,15]. In many compound datasets, both continuous and discontinuous SARs are found to coexist that are formed by different compound subsets [16]. The coexistence of locally continuous and discontinuous SARs gives rise to global SAR heterogeneity [16] and 3D ALs containing both smooth and rugged regions, termed variable ALs [14,15]. Of note, a 3D AL principally represents a non-linear quantitative SAR (QSAR) model, given its interpolated potency hypersurface to which descriptor coordinates of test compounds can be mapped. As such, the 3D AL is suitable for mapping of active compounds to regions of high or low potency, but not for actual potency prediction in lieu of machine learning [15]. This typically is a consequence of intrinsic overfitting of a 3D AL model to a given dataset, which yields a high-resolution SAR visualization, but prohibits generalization of the model for the prediction of numerical potency values [15]. Hence, while numerical SAR analysis functions are applicable to account for SAR continuity [16], discontinuity [16,17,18], or heterogeneity [16] in datasets, 3D AL models have thus far only been qualitatively analyzed and compared [19].

While SAR visualization is a key task of 3D AL modeling, one would clearly benefit from a more quantitative comparison of 3D ALs. Qualitative analysis of 3D ALs typically aims at relating topological features to SAR characteristics such as the relative content of continuous vs. discontinuous SAR components. One of the key tasks in SAR exploration of compound datasets is revealing differences in SAR information between different sets [5]. For example, for practical compound optimization, one often would like to assess which sets of compounds with activity against related targets have similar SAR characteristics and are rich in SAR discontinuity. Such datasets would be preferentially selected as compound source to guide optimization efforts. On the other hand, for computational SAR modeling and QSAR, one would like to give preference to datasets that contain more SAR continuity. Estimating and comparing relative SAR information content goes beyond the opportunities of SAR visualization and qualitative AL comparison. For example, large-scale SAR analysis would greatly benefit from identifying datasets that have similar SAR characteristics to a given compound set of interest, which is impossible on the basis of visual inspection. Hence, the ability to systematically relate topological differences to varying SAR information content and quantify SAR similarity of different datasets would complement SAR visualization and further extend the utility of 3D ALs, beyond intuitive analysis and comparison. Quantitative assessment would also aid in differentiating between datasets with heterogeneous 3D ALs displaying subtle topological differences that are difficult to appreciate on the basis of visual inspection. This is of practical relevance when evaluating the potential of further advancing SARs for compounds with activity against related targets. In such cases, one would favor focusing on compound sources capturing more SAR discontinuity than others, similar to the application scenario described above. Hence, there are multiple reasons motivating the development of methods for quantitative comparison of ALs.

However, from a computational point of view, the development of quantitative 3D AL methods is far from being a trivial task. Recently, it has been attempted to classify 3D ALs using machine learning (ML) based on features extracted from color-coded AL images using convolutional neural networks [20]. For a given 3D AL, variants with altered topologies were generated by either increasing the smoothness (continuity) or ruggedness (discontinuity) of the original AL. These topologically modified reference states were then distinguished from original 3D ALs by binary class label prediction using various ML approaches including deep learning [20]. These studies provided first evidence that 3D AL models with different topological features can be correctly classified on the basis of image data. However, the findings were limited to 3D AL variants and reference states with deliberately modified topologies. Accordingly, it remained unclear whether image processing might also be applicable to differentiate between original heterogeneous 3D ALs. Therefore, we have investigated a conceptually different image-based computational approach to determine (dis)similarity relationships between original 3D ALs of different compound datasets.

## 2. Results and Discussion

### 2.1. Activity Landscape Images

Images generated from 3D ALs preserve pairwise compound similarity relationships and their potency data as topographical features. Topology and color codes account for different SAR characteristics of compound datasets. Of note, SARs are determined by potency differences between compounds with varying degrees of similarity and are thus largely independent of absolute potency values. The interpolated potency surface of a 3D AL yields color gradients that can be represented in heatmaps without significant information loss (see Methods) [20]. Such heatmaps represent a top-down view of the 3D AL and the encoded color gradients implicitly—but comprehensively—account for the spatial distribution of topological features. Hence, 3D ALs and corresponding heatmaps are in principle well suited for image analysis. Given the aim of our method development effort, we have reasoned that comparing AL image features in a well-defined way should have the potential to discriminate between different 3D ALs in quantitative terms.

### 2.2. Image Similarity Analysis

**Figure 1:**
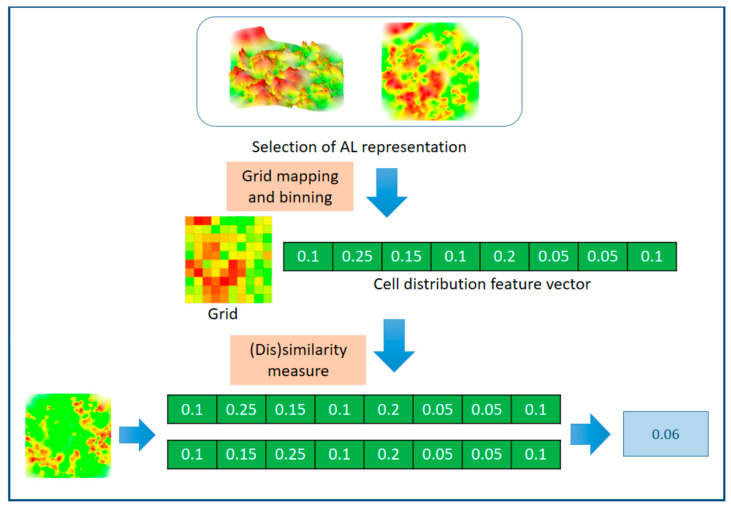
Three-dimensional activity landscape (AL) image-based similarity assessment. The schematic illustrates similarity analysis involving the conversion of 3D AL images into heatmaps, grid-based classification of heatmap cells according to different color intensity thresholds, and quantitative comparison of the resulting cell distribution feature vectors as a measure of AL (dis)similarity.

Three-dimensional AL images embed topological features and color profiles, which are characterized by different color gradients resulting from potency value and compound similarity distributions (the combination of which determines AL topology). In heatmaps derived from 3D ALs, topological features and ensuing color gradients are encoded by color pixel intensities that can be algorithmically extracted. The basic premise underlying similarity-based comparison of 3D AL images, as introduced herein, is that scaled color pixel intensities can be quantitatively compared across different heatmaps. To this end, a common grid representation of heatmaps plays a central role. Using an evenly spaced grid, the heatmap is divided into a constant number of cells, which are assigned to different categories based upon color intensity threshold values. The distribution of cells over different categories is then quantitatively compared as a measure of AL (dis)similarity. Figure 1 illustrates the approach. Methodological and calculation details are provided in the Methods Section.

### 2.3. Heatmaps and Grid Representations

**Figure 2:**
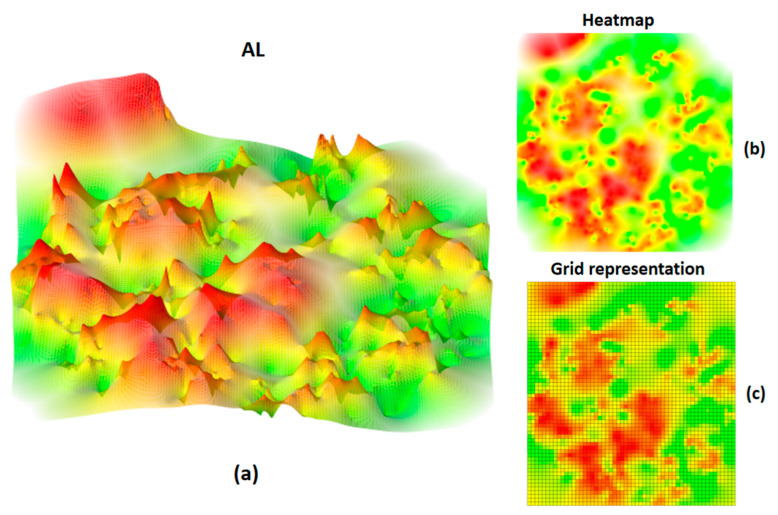
Activity landscape and heatmap representations. In (**a**), the 3D AL of a set of corticotropin-releasing factor receptor 1 ligands taken from ChEMBL version 23 [21] is shown (generated as detailed in the Methods Section). The surface is color-coded according to compound potency using a continuous spectrum ranking from red (high potency) over yellow to green (low potency). In (**b**), the corresponding heatmap is displayed. In (**c**), the heatmap is represented on an evenly spaced grid.

Conversion of 3D AL images into heatmaps established a reference frame for quantitative AL comparison. The heatmap corresponded to a top-down view of the color-coded 3D AL. Heatmaps were mapped onto an evenly spaced grid of dimensionality 56 × 60. Accordingly, each heatmap was divided into total 3360 cells. Figure 2 shows a 3D AL representation for a set of 673 corticotropin-releasing factor receptor 1 ligands, the corresponding heatmap, and its grid representation.

**Figure 3:**
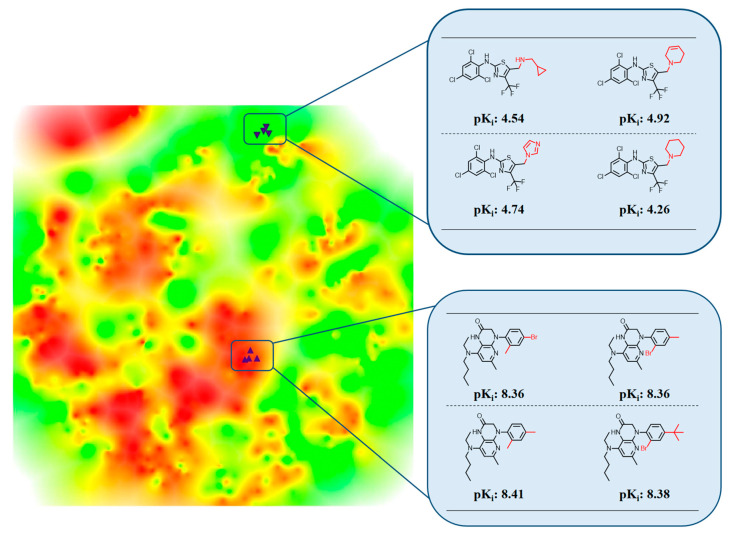
Heatmap with compound positions. The heatmap from Figure 2b is enlarged and positions of different compounds are indicated using black triangles. Exemplary weakly potent (top) and highly potent compounds (bottom) belong to two different analog series and map to green regions (valleys) and red regions, respectively. For each compound, its potency value is reported, and structural modifications distinguishing analogs from each series are highlighted in red.

In Figure 3 below, the heatmap of the of corticotropin-releasing factor receptor 1 ligands is enlarged, and positions of exemplary weakly or highly potent compounds are mapped. These compounds originated from two different analog series and occupy distant regions in the heatmap. The weakly potent compounds are found in a green region (corresponding to a valley) and the highly potent in a red region (formed by peaks). The representation illustrates color intensity-based encoding of 3D AL topology resulting from different compound potency levels.

For heatmaps, red and green channel pixel intensity values were combined into a single intensity value ranging from -1 to 1 (see Methods). To identify peaks using color intensities, positive threshold value intervals of (0, 0.25), (0.25, 0.5), (0.5, 0.75), and (0.75, 1.0) were applied. To identify smooth regions (valleys), negative threshold intervals of (0, −0.25), (−0.25, −0.5), (−0.50, −0.75), and (−0.75, −1.0) were used. Thus, different threshold intervals represented highest elevations (peaks), deepest valleys, and intermediate peak-to-valley and valley-to-peak regions. It should be noted that pixel intensities do not only encode potencies of individual molecules. Because intensities are obtained by interpolating a color gradient reflecting potencies of neighboring compounds, pixel intensities also implicitly account for locality information.

### 2.4. Grid-Based Similarity Analysis

Heatmap cells were assigned to eight different categories on the basis of the threshold value intervals specified above. The assignment yielded an AL-dependent distribution of categorized cells. Grid-based partitioning of a heatmap and categorization of the resulting cell population provided two fundamental advantages for subsequent similarity analysis. First, a constant number of cells was obtained; second, the distribution of cells over different threshold categories was image orientation-invariant. While grid locality information is not preserved in the global cell distribution, SAR information from neighboring molecules is retained by interpolated intensities and hence implicitly included in the comparison. As a measure of AL (dis)similarity, cell distributions of different heatmaps were quantitatively compared by calculating symmetric relational entropy and cosine distances (see Methods).

### 2.5. Activity Landscape Comparison

**Figure 4:**
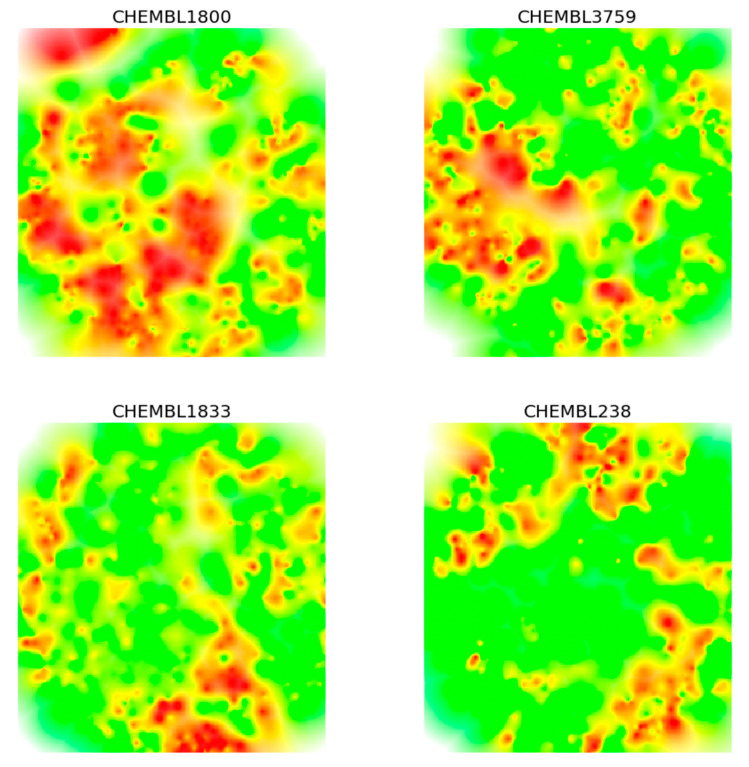
Heatmaps from different activity landscapes. Heatmaps derived from 3D ALs of four different compound datasets according to Table 1 are compared.

Figure 4 shows heatmaps for 3D ALs of four exemplary compound datasets from ChEMBL version 23 [21] that are reported in Table 1. The datasets consisted of 673–887 compounds with activity against different targets covering different potency ranges. All four sets were characterized by SAR heterogeneity, i.e., their 3D ALs contained both smooth and rugged regions, corresponding to SAR continuity and discontinuity, respectively. However, on the basis of visual inspection, there also were apparent differences between these ALs, reflecting varying SAR information content. For example, the heatmap of compound dataset CHEMBL1800 (C1800) contained more and more widely distributed peak regions than the others, and C1800 and CHEMBL238 (C238) appeared to be overall the most dissimilar pair. Other relationships involving CHEMBL3759 (C3759) CHEMBL1833 (C1833) were difficult to judge, illustrating the limitations of visual inspection.

**Figure 5:**
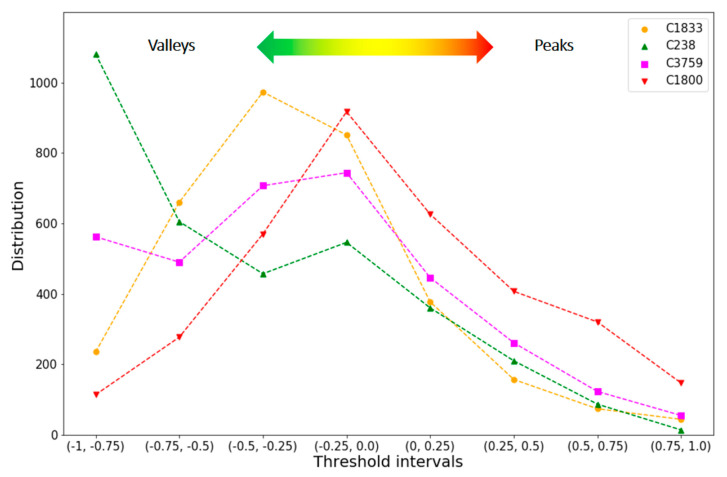
Cell intensity distributions. For the four compound datasets, cell distributions over eight threshold intervals are reported and color-coded as indicated. From left to right, valleys (interval 1–3), intermediate regions (interval 4,5), and peaks (interval 6–8) are accounted for.

For the heatmaps, we then determined the grid-based cell intensity distributions over the eight threshold intervals. Figure 5 compares these distributions.

Large differences between these distributions were found in smooth regions. Here, C238 (green curve in Figure 5) had by far the largest number of cells accounting for valleys and C1800 (red) the smallest. Furthermore, C1800 and C238 had the largest and smallest number of cells covering intermediate regions, respectively. In peak regions, the distributions of three of the four datasets were similar, except C1800, which had a larger number of cells accounting for peaks than the others. These findings were consistent with conclusions that could be drawn from visual inspection. Going beyond what could be concluded on the basis of visual inspection, the profiles of C1833 (orange) and C3759 (magenta) were found to be overall similar. While C1833 had more cells accounting for smooth regions than C3759, the traces of the distributions closely followed each other in intermediate and peak regions. Taken together, comparison of cell intensity distributions revealed quantifiable differences between AL images of different datasets and thus provided a sound basis for (dis)similarity analysis.

To quantify differences between cell distributions in a pairwise manner and provide a numerical measure of AL (dis)similarity, relative entropy (RE) was determined by calculating the Kullback–Leibler divergences (KLD) [22] between feature vectors of cell distributions (see Methods). In addition, cosine distance (CD) values were determined for pairwise comparison of distribution feature vectors, a standard dissimilarity measure [23]. Increasing RE and CD values indicate increasing dissimilarity between ALs. Table 2 reports the results of pairwise comparisons of the cell intensity profiles.

As expected, largest RE (0.53) and CD (0.33) values were obtained for the C1800/C238 comparison, confirming that the ALs of these datasets were most dissimilar. By contrast, smallest RE and CD values were calculated for the C3759/C1833 (0.08 and 0.05, respectively) and the C3759/C238 comparison (0.09 in both instances). As discussed above, C3759 and C1833 yielded the overall most similar cell distributions. Furthermore, for the C1833/C238 comparison, intermediate RE (0.28) and CD (0.24) values were obtained, which were also reconcilable on the basis of the observed distribution traces. The comparisons revealed that RE and CD calculations were suitable for comparing cell distribution feature vectors. Since RE values covered a larger value range for the reported comparisons than CD values, we would assign preference to the former, at least in these cases. Regardless, the calculations reported herein are generally applicable and provide a first quantitative measure of 3D AL (dis)similarity.

### 2.6. Conclusions

The AL concept was introduced for graphical analysis of SARs contained in compound datasets. For SAR visualization, 3D AL representations are particularly intuitive since they are akin to geographical maps and their topological features mirror SAR characteristics. Three-dimensional ALs of most compound datasets are variable in nature, reflecting different degrees of SAR heterogeneity. Going beyond visual inspection and qualitative comparison of 3D ALs, the ability to quantitatively account for topological differences between 3D ALs would provide substantial support for SAR exploration of compound datasets and various practical applications. In this work, we have introduced a computational methodology to quantify (dis)similarity relationships between 3D ALs on the basis of image data. Three-dimensional AL images can be converted into heatmaps representing a top-down view of the ALs with very little loss in information such that color intensities and textures represent topological features. Heatmaps are then mapped onto evenly spaced grids with constant dimensions, which yields a constant number of cells, providing a basis for AL comparison. These cells are then categorized on the basis of color intensities, which implicitly account for the spatial distribution of corresponding topological features they represent. Differences in the distribution of cells over different threshold intervals are then quantified as a measure of 3D AL (dis)similarity. Importantly, cell-based comparison of ALs is image-orientation invariant and thus generally applicable. As shown in our proof-of-concept investigation, comparison of categorized cell distributions provides a meaningful quantitative readout for comparison of 3D ALs and, thus, further extends the utility of AL representations for SAR exploration.

## 3. Methods

### 3.1. Three-Dimensional Activity Landscapes

Three-dimensional AL models of compound datasets (Figure 2) were generated following the protocol reported in [20]. Briefly, chemical reference space was generated on the basis of extended connectivity fingerprint with bond diameter 4 (ECFP4) [24] Tanimoto distances [25] for pairwise compound comparisons. The 2D projection of chemical reference space was computed using multi-dimensional scaling (MDS) [26], applying a stress function based on pairwise Tanimoto distances. The potency surface was interpolated via Gaussian process regression (GPR) [27] and color-coded by compound potency applying a continuous color gradient from red (highest potency in a dataset) over yellow to green (lowest potency). Intermediate potency values were computed using a Gaussian process based on prior covariances of experimental potency values. The “Sum of Matern and White” kernel [27] was used assuming a mean of zero to derive relationships between experimental data points (potency values). Gaussian noise factors were applied to permit minor variations of z-values for points on the x-y plane and optimize the global fit of the surface to experimental data points. Noise factors were adjusted for each target activity class by optimizing the kernel’s alpha parameter between 10^−1^ and 10^−7^ over 10 iterations. The gradient was applied to a limited pK_i_ range from 3.72 (green) over 5.75 (yellow) to 8.75 (red). Potencies outside this range were assigned to green (less than 3.72) or red (larger than 8.75).

### 3.2. Image Processing and Analysis

For each 3D AL, a heatmap was initially obtained using the RGB color model of OpenCV version 3.0 with eight bits per channel [28,29]. Heatmaps were cropped to dimensions of 280 × 300 pixels (starting from the original 600 × 400 pixels including white excess areas). Because 3D AL models were created by interpolating potency values using the color gradient from red over yellow to green, without using the blue channel, the red and green (RG) channel pixel values were extracted by subtracting green channel intensity values from red channel intensity values and combined into a single intensity value ranging from −255 to 255. Accordingly, the dataset compound with lowest potency (brightest green pixels), intermediate (yellow pixels), and highest potency (brightest red pixels) corresponded to values of −255, 0, and +255, respectively. RG pixel values were then normalized to the range of −1 to +1. The RG color model preserved more than 95% of the RGB colors, except for shades of white (i.e., interpolated surface area without experimental potency), which were accounted for by yellow hues using the RG model.

### 3.3. Grid Representation

Each heatmap was mapped to an evenly spaced grid of dimensionality 56 × 60, forming total 3360 square cells. Color intensity values were divided into eight different threshold intervals (categories), as specified above. Average pixel intensity values from the 25 pixels of each cell were assigned to the cell, and the distribution of cells over the eight threshold intervals was determined. For comparison, cell intensity distributions were encoded as individual feature vectors.

### 3.4. Similarity Analysis

To quantify (dis)similarity between any two 3D ALs images based upon their heatmaps, relative information entropy and cosine distances were calculated for cell distribution feature vectors. Relative entropy (RE), also known as the Kullback−Leibler divergence (KLD), is calculated between two probability distributions *P*(x), *P*(y). These were obtained from the feature vectors x and y by converting the distributions to relative frequencies. KLD is defined as [22]:

(1) $$KLD\left(P\left(x\right)||P\left(y\right)\right)=\;\sum^{\;}P\left(x\right)\;log\left(\frac{P\left(x\right)}{P\left(y\right)}\right)\;\;$$

Given the intrinsic asymmetry of KLD, symmetric relative entropy values for comparison of feature vector probability distributions *P*(*x*) and *P*(*y*) were obtained by taking the average of KLD(P(x)||P(y)) and KLD(P(y)||P(x)):

(2) $$RE\left(P\left(x\right),P\left(y\right)\right)\;=\;\frac{\sum^{\;}P\left(x\right)\;log\left(\frac{P\left(x\right)}{P\left(y\right)}\right)\;+\sum^{\;}P\left(y\right)\;log\left(\frac{P\left(y\right)}{P\left(x\right)}\right)\;\;}{2}$$

In addition, cosine distances between feature vectors were calculated. The cosine coefficient is widely used to measure the relationship between any two given feature vectors by calculating the cosine of the angle between the two vectors [23]. It is defined as the inner product of two vectors divided by the product of their lengths. The cosine distance CD is obtained by subtracting the cosine similarity value from 1 and given by:

(3) $$CD\left(x,y\right)=1-\;\left(\frac{x\cdot y}{\left\|x\|\|y\right\|}\right)$$

## Figures and Tables

**Table 1:** Datasets. The table summarizes the composition of four exemplary compound datasets (different activity classes) used for 3D AL analysis.

ChEMBL Target ID	Target Name	Number of Compounds	Potency (pK_i_)	
			Min	Max
1800	Corticotropin-Releasing Factor Receptor 1	673	4.3	9.7
3759	Histamine H4 receptor	887	2.9	10.4
1833	5-hydroxytryptamine receptor 2B	695	5.0	10.0
238	Sodium-dependent dopamine transporter	850	2.1	9.4

**Table 2:** Similarity calculations. Relative entropy (RE) and cosine distance (CD) values for comparison of cell distribution feature vectors are reported.

AL Comparison	RE	CD
C1800/C3759	0.19	0.10
C1833/C238	0.28	0.24
C1800/C1833	0.22	0.12
C1800/C238	0.53	0.33
C3759/C1833	0.08	0.05
C3759/C238	0.09	0.09
